# Association between multiple dimensions of access to care and cervical cancer screening among Kenyan women: a cross-sectional analysis of the Demographic Health Survey

**DOI:** 10.1186/s12913-024-11169-8

**Published:** 2024-06-14

**Authors:** Xiaowan Li, Sanmei Chen, Naoki Hirose, Yoko Shimpuku

**Affiliations:** 1 Department of Pediatric Hematology and Oncology Nursing, West China Second University Hospital, Sichuan University, Chengdu, Sichuan China; 2 Key Laboratory of Birth Defects and Related Diseases of Women and Children (Sichuan University), Ministry of Education, Chengdu, Sichuan China; 3 Global Health Nursing, Graduate School of Biomedical and Health Sciences, Hiroshima University, Hiroshima, Japan

**Keywords:** Access to care, Cervical cancer screening, Kenya, Sub-Saharan Africa

## Abstract

**Background:**

Cervical cancer remains the second most common cause of death in women and poses a growing public health challenge. It is urgent to increase cervical cancer screening rates in Kenya as per the 2018 Kenya National Cancer Screening Guidelines. Addressing access to care may serve as a target to achieve this goal; however, how individual dimensions of access to care are associated with the utilization of cervical cancer screening services in low- and middle-income countries, including Kenya, remains unclear. This study aimed to examine how different aspects of access to care (affordability, availability, geographical access, and social influence) were associated with cervical cancer screening among Kenyan women of reproductive age.

**Methods:**

This cross-sectional study used data from the 2014 Kenya Demographic and Health Survey and the 2010 Kenya Service Provision Assessment. The final sample included 5,563 women aged 25–49 years. Logistic regression models were used to examine the association between different aspects of access to care and the uptake of cervical cancer screening.

**Results:**

Factors such as being in the poorest wealth quintile, lacking health insurance, having difficulty obtaining funds for treatment (affordability), limited availability of screening services at nearby facilities (availability), living in rural areas (geographical access), and having healthcare decisions made solely by husbands/partners or others (social influence) were associated with a decreased likelihood of the uptake of cervical cancer screening.

**Conclusions:**

Increasing health insurance coverage, enhancing the availability of screening services at health facilities, expanding mobile screening health facilities in rural areas, and empowering women to make their own healthcare decisions are crucial steps for increasing cervical cancer screening uptake in Kenya.

**Supplementary Information:**

The online version contains supplementary material available at 10.1186/s12913-024-11169-8.

## Introduction

Cervical cancer is the second leading cause of death among women globally and poses a growing public health challenge [1]. In 2020, there were approximately 604,000 new cervical cancer cases and an estimated 342,000 deaths from the disease worldwide, 84–88% of which occurred in low-resource African regions [2]. Africa, particularly Sub-Saharan Africa (SSA), has the highest rates of cervical cancer morbidity and mortality [1–3]. Regular screening and early treatment are crucial for preventing cervical cancer [4–6]. However, in Kenya, a SSA country, the cervical cancer screening rate was only 16.4% in 2015, largely because of limited access to screening services [7]. The 2018 Kenya National Cancer Screening Guidelines (KNCSG) by the Ministry of Health, recommends an increase in cervical cancer screening rates among women in the 25–49 age group to identify and treat cervical cancer earlier [7]. Thus, improving cervical cancer screening rates in Kenya is critical.

In Kenya, studies have investigated the risk and protective factors for cervical cancer screening, such as risky sexual behaviors and sociodemographic factors (e.g., age, level of education, religion, HIV screening, exercise, etc.) [8–12]. The 2018 KNCSG underscore the importance of public health initiatives in promoting cervical cancer screening; enhancing access to care is a first step in boosting screening uptake [7]. 

Access to care, a functional link between the population, health facilities, and medical resources, encompasses several dimensions such as affordability, availability, geographical access, accommodation, and acceptability [13]. Additionally, the concept of “social influence,” which refers to how social and cultural factors affect healthcare-seeking behavior, has been recognized for its relative importance [14]. Moreover, previous research does not focus on the different dimensions of access to care. (14–15) The way in which multiple dimensions of access to care are associated with cervical cancer screening in low- and middle-income countries, including Kenya, remains unclear.

This study explored the association between various aspects of access to care and cervical cancer screening among Kenyan women of reproductive age.

## Methods

### Data

This study used data from the 2014 Kenya Demographic and Health Survey (KDHS) and the 2010 Kenya Service Delivery Assessment (KSPA). The 2014 KDHS, a household survey aligned with international standards, gathered data on a wide range of topics, including demographics, socioeconomic factors, and health, from a nationally representative sample of Kenyan households. The KDHS is globally recognized for providing reliable data on various aspects, such as age, religion, education, HIV testing, and physical activity [6, 17]. The 2014 KDHS sample was taken from the Fifth National Sample Survey and Evaluation Program, which served as the master sampling framework [16, 17]. A two-stage sampling design was used for each stratum, where 1,612 clusters were selected with equal probability from the frame of the Fifth National Sample Survey and Evaluation Program in the first stage, and 25 households were subsequently selected from each cluster in the second stage. Of these, 1,594 clusters were confirmed to be occupied [16]. The 2014 KDHS data provided Global Positioning System (GPS) data of each participant’s home.

**Fig. 1:**
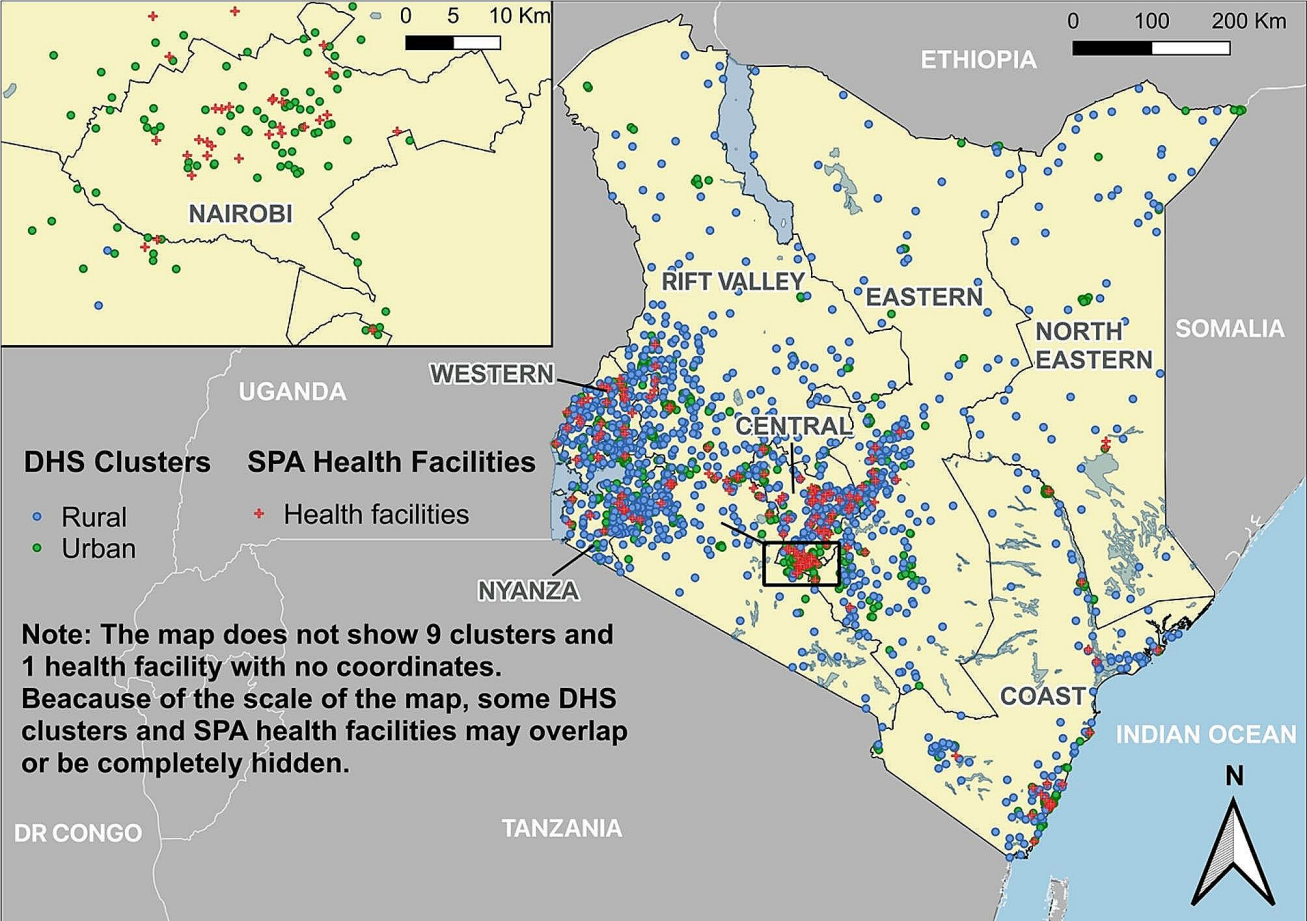
The geographic location of Demographic and health survey clusters (displaced) and service provision assessment facilities Abbreviations: DHS: Demographic and Health Survey; SPA: Service Provision Assessment; yellow section; Kenyan territory; the blue dots represent rural clusters; and the green dots represent urban clusters. The red dots represent health facilities. The map does not show the nine clusters or one health facility with no coordinates. Owing to the scale of the map, some DHS clusters and SPA health facilities may overlap or become completely hidden. To increase the readability of the distributions of SPA health facilitators and DHS health facilitators, Nairobi was zoomed out as an example. To protect privacy, the coordinates provided to the researcher by the DHS program were displaced within a certain range of real coordinates

The sample of the 2010 KSPA was carefully designed to showcase essential indicators according to the facility type and various regulatory authorities. The final KSPA sample represented approximately 11% of all facilities in the country (National Coordinating Agency for Population and Development et al., 2011) [18]. Data were collected from 695 health facilities, resulting in a 99% success rate in data collection. Of these facilities, 76 (when weighted) provided at least one method of cervical cancer screening, as shown in Fig. 1 (administrative units from before 2010 to be consistent with the 2014 KDHS). The 2010 KSPA data provided GPS data of each facility. All GPS data provided by the DHS program were intentionally displaced to a certain extent to safeguard national coordinate information.

**Fig. 2:**
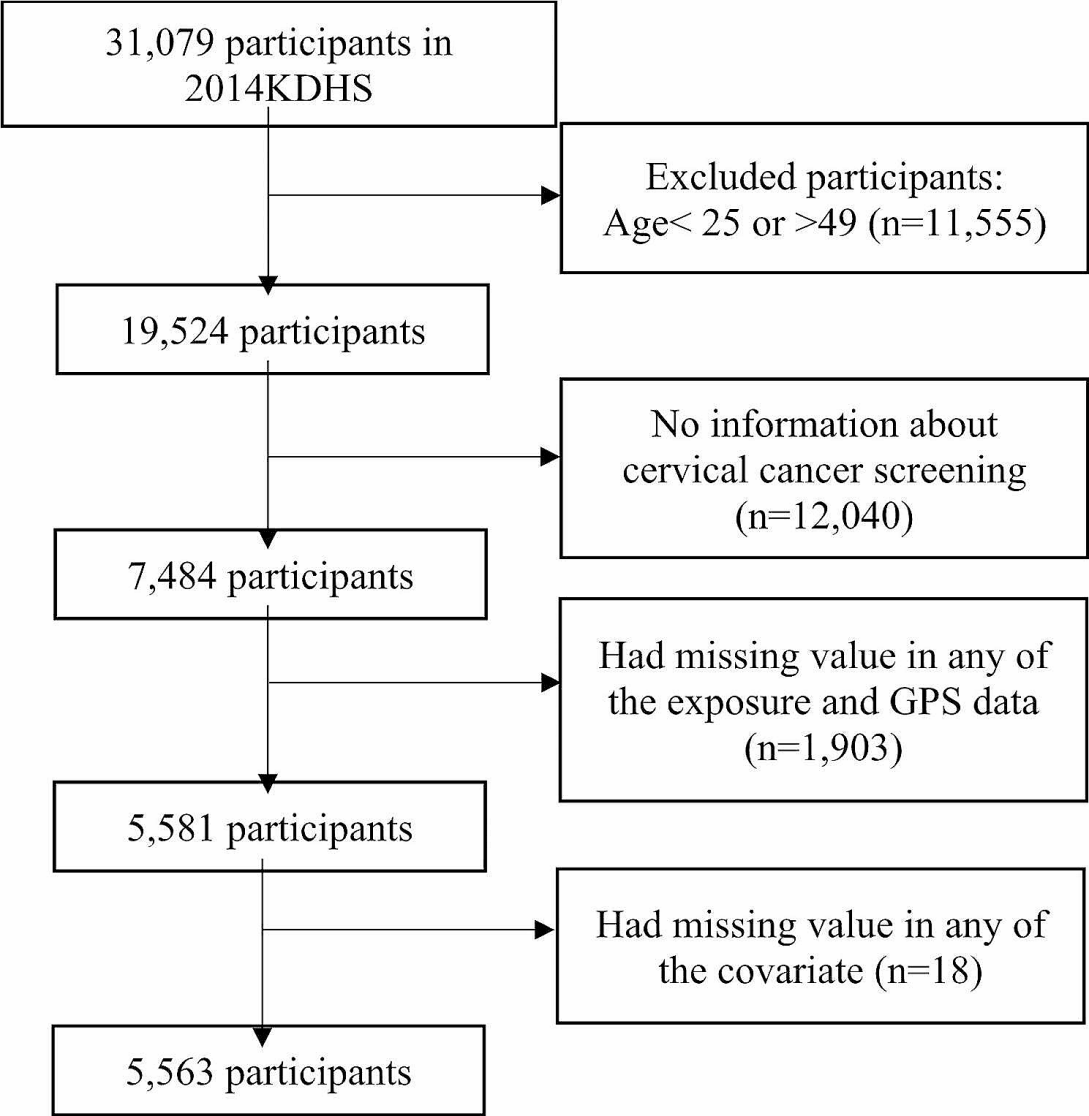
Flowchart of the study population selection Abbreviations: KDHS: Kenya Demographic and Health Survey; GPS: Global Positioning System

Of the 31,079 women in the 2014 KDHS, we focused on the 25–49 years age group as per the 2018 Kenya National Cancer Screening [7], excluding those outside this range (*N* = 19,524). We excluded women with incomplete cervical cancer screening data (*N* = 7,484), missing GPS data (*N* = 5,581), and a lack of exposure and covariate information (*N* = 5,563) (Fig. 2). The final weighted sample for the statistical analysis comprised 5,563 women across 1,594 clusters.

### Exposure

We defined the following four dimensions of access to care: affordability, availability, geographical access, and social influence based on the “5 As of access” criteria by Penchansky & Thomas and Moyer et al. (Table 1) [13, 14]. We did not define the dimensions of accommodation, and acceptability due to the limitations of the 2014 KDHS data.

**Table 1:** The “5 as of access” framework, as assessed in the 2014 Kenya demographic health survey and 2010 Kenya service provision assessment

5 As of access category	Definition	DHS/SPA item
Affordability	How the provider’s charges relate to the patient ability and willingness to pay for services	Wealth index (KDHSHQ102,107,110:c,118: b,118 A,118B) Covered by health insurance (KDHSWQ1009) Barrier to getting the money needed for medical advice or treatment (KDHSWQ1008b)
Availability	A measure of the extent to which providers have the medical resources to deliver the needs of their patients	Level of availability of cervical cancer screening services in any health facility in the sampling stratum where each participant lived (KSPAQ570)
Geographical access	Geographic accessibility	Distance to health facility as a barrier to get medical advice or treatment (KDHSWQ1008c) Rural, urban residence (KDHSWQ: IDENTIFICATION) Region of residence (KDHSWQ: IDENTIFICATION) Healthcare facility visit past 12 months (KDHSWQ327)
Accommodation	Degree to which providers are patient-centered in their operations	^a^
Acceptability	Patient satisfaction with health care providers	^a^
Social influence	Extent of influence of social and cultural factors on care seeking behavior	Needing permission to seek healthcare (KDHSWQ1008a) Not wanting to go to health facility alone (KDHSWQ1008d) Who has the final say in healthcare decisions (KDHSWQ820)

KDHSHQ: household questionnaire from the 2014 Kenya Demographic and Health survey

KDHSWQ: women’s questionnaire from the 2014 Kenya Demographic and Health survey

KSPAQ: service Provision Assessment questionnaire from the 2010 Kenya Service Provision Assessment

^a^: Lack of relevant questionnaire

#### Affordability

Affordability was assessed using three indicators: (1) household wealth index levels (categorized as poorest, poor, middle, richer, and richest); (2) whether being covered by health insurance (yes or no); and (3) the extent to which obtaining money for medical advice or treatment was a problem (not a big problem, or a big problem). The wealth index is a composite measure derived from household assets, such as televisions and bicycles, building materials, types of water access, sanitation facilities, and other wealth-related characteristics [16]. The DHS program uses principal component analysis to standardize these asset factor scores into five wealth quintiles (poorest, poor, middle, rich, and richest) [16]. 

#### Availability

We defined availability of cervical cancer screening services accordingly to the number of healthcare facilities providing at least one type of cervical cancer screening test including pap smear, HPV test, and visual inspection with acetic acid within the residential area of each participant. This definition was operationalized by using both of the 2014 KDHS GPS data and the 2010 KSPA GPS data. To minimize the potential classification bias of availability, we used a clustering method as recommended by the DHS when quantifying the number of nearest facilities with available cervical cancer screening [18]. This clustering method considered the setting of service as well as the displacement of coordinates, rather than simply calculating the geographic distance of the healthcare facility from the cluster. Specifically, we employed a buffer zone strategy, as recommended by the DHS guidelines [18]. We firstly verified the origins of GPS data and coordinates for all surveyed health facilities and clusters where each participant was living. We then excluded nine clusters lacking coordinate data (seven rural and two urban) from the analysis. As we mentioned above, participants in those excluded clusters were excluded from the final sample. Additionally, one health facility without coordinates was treated as having missing data and was excluded. We merged these datasets using GPS coordinates to create a buffer zone around each cluster using a 5 km range from the center of urban clusters and a 10 km range from the center of rural clusters [16, 19–24]. Finally, 1,585 buffer zones were created (615 urban and 970 rural). We then counted the number of healthcare facilities with available cervical cancer screening within each buffer zone.

Availability was then determined by the number of healthcare facilities within the buffer in which each participant lived. Based on this number, the availability level was then categorized as low (no facilities), medium (1 facility), or high (≥ 2 facilities).

We also identified the types of health facilities providing screening services, such as national referral hospitals, provincial hospitals, district hospitals, subdistrict hospitals, other hospitals, health centers, clinics, dispensaries, and maternities, for descriptive purposes.

#### Geographical access

We used four indicators to define geographical access: (1) distance to health facility as a barrier to get medical advice or treatment (categorized as “not a big problem” or “big problem”); (2) type of residence (rural or urban); (3) whether the individual visited healthcare facilities in the past 12 months (yes or no), and (4) region of residence (Coast, Eastern, North-Eastern, Central, Rift Valley, Western, Nyanza, and Nairobi as recommend by the KDHS program). Due to the small number of women in the North-Eastern region, we combined Eastern and North-Eastern.

#### Social influence

The dimension of social influence was measured using three indicators: (1) barrier in needing permission to seek healthcare (not a big problem or a big problem); (2) not wanting to go to health facility alone (not a big problem or big problem ); and (3) decide on health care. Decision makers were categorized into three groups: the woman alone, the woman and her husband/partner jointly, or husband/partner or another individual alone.

### Outcome

The 2014 KDHS assessed the prevalence of cervical cancer screening by asking participants, “Have you ever been tested or examined for cervical cancer?” with possible responses being “yes,” “no,” or “don’t know.” In our analysis, a “no” response was coded as “1,” a “yes” response as “0,” and “don’t know” responses were considered missing data.

### Covariates

In this study, covariates that have been shown to affect cervical cancer screening were used as covariates. The covariates selected for this study are based on data availability from previous studies and the 2014 KDHS dataset. Age was considered a continuous variable. Religion was classified as Roman Catholic, Protestant, Other Christians, or Muslim according to the responses from the self-report questionnaire. Additional variables included educational level (none, primary, secondary, or higher), HIV screening (yes/no), physical exercise (yes/no), and frequency of engaging with media, such as reading the newspaper, listening to the radio, or watching TV, categorized as either not at all/less than once a week or at least once a week.

### Data analysis

We used descriptive statistics to summarize the sociodemographic characteristics, presenting categorical variables as proportions and continuous variables as the means and standard deviations (SD).

Logistic regression models were used to estimate the association between the access to care dimensions and cervical cancer screening. Given the KDHS and KSPA’s stratified two-stage cluster sampling, we conducted a weighted logistic analysis to reduce bias and enhance precision (Kenya National Bureau of Statistics, Ministry of Health et al., 2014) [7, 16]. This analysis proceeded after confirming that there was no significant multicollinearity among the independent variables (variance inflation factor < 10), and the absence of significant outliers. First, we created an unadjusted crude model, followed by a second model adjusted for the education level. The final model included adjustments for all covariates: age; religion; education; HIV screening; physical exercise; and frequency of engagement with newspapers, radio, and television. The Hosmer–Lemeshow test verified the good fit of all the models, with *p* values above 0.05.

The threshold for statistical significance was set at *p* < 0.05. In line with KDHS guidelines [17], all analyses were performed to account for unweighted and complex sampling conditions. SPSS Statistics software (version 28; IBM, Armonk, NY, USA) was used for the analysis. Additionally, a medical situation map for Kenya was created using the open-source software QGIS (version 3.28.0; OSGeo, USA).

## Results

Table 2 shows the sociodemographic and health-related characteristics of the participants. The average age was 34.3 years, with a standard deviation of ± 6.8 years. Among the 5,563 participants in the final sample, 76.7% had never undergone cervical cancer screening.

**Table 2:** Sociodemographic characteristics of a weighted sample from the 2014 Kenya demographic health survey

Variables	Total	Cervical cancer screening	
	*n* (%)	No (%)	Yes(%)
Weighted sample	5563 (100)	4268 (76.7)	1295 (23.3)
**Age (years) Mean(SD)**	34.3 (6.8)	34.1 (6.8)	35.1 (6.7)
**Religion**			
Roman Catholic	1126 (20.2)	819 (72.7)	307 (27.3)
Protestant/Other Christian	4153 (74.7)	3204 (77.1)	949 (22.9)
Muslim	213 (3.8)	187 (87.8)	26 (12.2)
Other	71 (1.3)	57 (80.3)	14 (19.7)
**Highest educational level**			
No education	213 (3.8)	191 (89.7)	22 (10.3)
Primary	3050 (54.8)	2442 (80.1)	608 (19.9)
Secondary	1599 (28.7)	1215 (76.0)	384 (24.0)
Higher	701 (12.6)	419 (59.8)	282 (40.2)
**HIV screening**			
No	291 (5.2)	258 (88.7)	33 (11.3)
Yes	5271 (94.8)	4009 (76.1)	1262 (23.9)
**Physical exercise**			
No	4362 (78.4)	3376 (77.4)	986 (22.6)
Yes	1202 (21.6)	892 (74.2)	310 (25.8)
**Frequency of reading newspaper or magazine**			
Not at all	3508 (63.1)	2862 (81.6)	646 (18.4)
Less than once a week	1154 (20.7)	823 (71.3)	331 (28.7)
At least once a week	901 (16.2)	583 (64.7)	318 (35.3)
**Frequency of listening to radio**			
Not at all	735 (13.2)	599 (81.5)	136 (18.5)
Less than once a week	639 (11.5)	508 (79.5)	131 (20.5)
At least once a week	4189 (75.3)	3161 (75.5)	1028 (24.5)
**Frequency of watching television**			
Not at all	2481 (44.6)	2098 (84.6)	383 (15.4)
Less than once a week	742 (13.3)	568 (76.5)	174 (23.5)
At least once a week	2339 (42.1)	1601 (68.4)	738 (31.6)

**Fig. 3:**
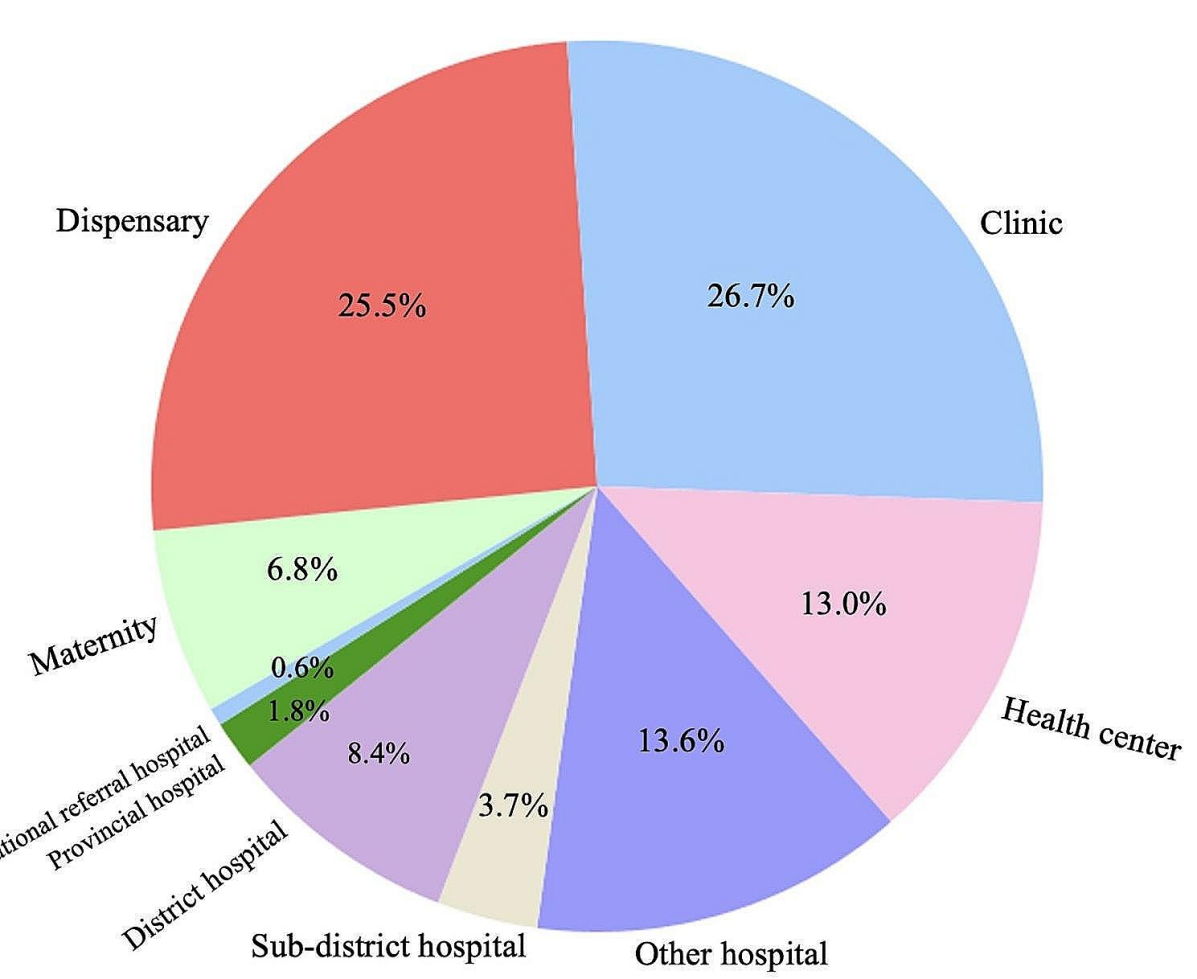
Percentage of institutions providing cervical cancer screening services by type The Pie chart indicate the percentage of individuals in each health facility type among the health facilities offering cervical cancer screening services

Figure 3 shows the proportion of facilities offering at least one form of cervical cancer screening. Clinics were the most prevalent providers, accounting for 26.7%, with dispensaries accounting for close to 25.5%. Other hospitals provided 13.6% of the screenings: health centres, 13.0%; district hospitals, 8.4%; maternity homes, 6.8%; subdistrict hospitals, 3.7%; provincial hospitals, 1.8%; and national referral hospitals, 0.6%.

Table 3 shows the association between the different dimensions of access to care and no cervical cancer screening. Regarding affordability, the final model showed that women in the poorest group were 2.71 times more likely to forego screening than those in the richest group (adjusted odds ratio (AOR) [95% CI]: 2.71 [1.71–4.29]). Uninsured women were 1.92 times more likely to not be screened than their insured counterparts were (AOR [95% CI]: 1.92 [1.55–2.37]). Additionally, women who perceived cost as a barrier to treatment and medical care had a 1.24 times greater chance of not receiving screening (AOR [95% CI]: 1.24[0.99–1.56]).

**Table 3:** Logistic regression analysis of access to care associated with factors associated with no screening

Variables	Crude OR (95%CI)	Education-adjusted OR (95%CI)	Fully-adjusted OR (95%CI)*
**Affordability**			
Wealth index			
Richest	Reference	Reference	Reference
Richer	1.81 (1.44–2.28)	1.65 (1.32–2.07)	1.47 (1.14–1.89)
Middle	2.64 (2.07–3.38)	2.30 (1.75–3.02)	1.94 (1.41–2.65)
Poorer	3.17 (2.49–4.03)	2.67 (2.02–3.52)	2.13 (1.52–2.99)
Poorest	4.37 (3.10–6.16)	3.49 (2.36–5.17)	2.71 (1.71–4.29)
Health insurance			
Yes	Reference	Reference	Reference
No	2.66 (2.22–3.18)	2.21 (1.80–2.72)	1.92 (1.55–2.37)
Barrier in getting money needed for medical advice or treatment			
Not a big problem	Reference	Reference	Reference
Big problem	1.66 (1.35–2.04)	1.41 (1.13–1.76)	1.24 (0.99–1.56)
**Availability**			
Level of availability of cervical cancer screening services			
High availability	Reference	Reference	Reference
Medium availability	1.61 (1.17–2.21)	1.50 (1.08–2.09)	1.39 (1.00-1.92)
Low availability	2.06 (1.52–2.78)	1.84 (1.34–2.53)	1.64 (1.20–2.26)
**Geographical access**			
Distance to health facility as a barrier to get medical advice or treatment			
Not a big problem	Reference	Reference	Reference
Big problem	1.51 (1.20–1.89)	1.32 (1.04–1.68)	1.18 (0.92–1.50)
Place of residence			
Urban	Reference	Reference	Reference
Rural	1.70 (1.41–2.05)	1.43 (1.16–1.76)	1.25 (1.00-1.57)
Region			
Nairobi	Reference	Reference	Reference
Eastern and North Eastern	2.26 (1.51–3.39)	1.94 (1.27–2.97)	1.88 (1.22–2.89)
Central	1.21 (0.81–1.81)	1.12 (0.74–1.69)	1.19 (0.79–1.79)
Rift Valley	2.29 (1.56–3.35)	2.10 (1.42–3.13)	2.02 (1.36-3.00)
Western	3.13 (2.03–4.82)	2.76 (1.76–4.32)	2.52 (1.60–3.97)
Nyanza	2.23 (1.51–3.30)	1.99 (1.33-3.00)	1.84 (1.21–2.80)
Coast	2.98 (1.96–4.54)	2.46 (1.57–3.85)	2.59 (1.66–4.06)
Healthcare facility visit past 12 months			
Yes	Reference	Reference	Reference
No	0.90 (0.69–1.19)	0.90 (0.68–1.19)	0.90 (0.67–1.19)
**Social influence**			
Barrier in needing permission to seek healthcare			
Not a big problem	Reference	Reference	Reference
Big problem	1.12 (0.78–1.61)	1.01 (0.69–1.47)	0.92 (0.63–1.35)
Not wanting to go to health facility alone			
Not a big problem	Reference	Reference	Reference
Big problem	1.27 (0.95–1.71)	1.19 (0.88–1.61)	1.07 (0.78–1.45)
Decide on health care			
Women alone and	Reference	Reference	Reference
Women	0.97 (0.80–1.19)	1.00 (0.81–1.23)	1.01 (0.82–1.24)
husband/partner			
Husband/partner or someone else alone	1.57 (1.24–1.98)	1.42 (1.12–1.79)	1.36 (1.07–1.73)

Note. CI: Confidence interval; OR: Odds Ratio

The fully adjusted model was adjusted for age, religion, education, HIV screening, physical exercise, frequency of reading newspapers or magazines, frequency of listing to the radio, and frequency of watching television

Concerning availability, those living in areas with low availability of cancer screening services were significantly more likely to have never been tested for cervical cancer (AOR [95% CI]: 1.64[1.20–2.26]).

In terms of geographical access, in the final model, rural residents faced higher odds of never being tested than urban residents (AOR [95% CI]: 1.25 [1.00–1.57]). Residents of coastal (AOR [95% CI]: 2.59 [1.66–4.06]) and western areas (AOR [95% CI]: 2.52 [1.60–3.97]) were significantly less likely to have never been tested than were those living in Nairobi.

For the social influence dimension, women who could not make their own healthcare decisions were significantly more likely to have never been tested (AOR [95% CI]: 1.36[1.07–1.73]). No significant associations were found for distance to health facilities, the need for permission to seek healthcare, or reluctance to visit health facilities alone as barriers to obtaining medical advice or healthcare facility visits.

## Discussion

In our study, we discovered that factors such as affordability (wealth index, health insurance, and financial barriers to medical advice or treatment), availability (access to cervical cancer screening services), geographical access (residence, region), and social influence (healthcare decision-making) were significantly associated with cervical cancer screening uptake.

We observed that affordability is positively associated with cervical cancer screening among women aged 25–49 years in Kenya. These findings align with those of a study in Kenya, which revealed that additional expenses, such as transportation and medical costs, hindered poor women from being screened [11]. However, these results contrast with another study conducted in South African, which found no significant link between wealth and screening rates [25]. The difference in healthcare systems between Kenya and South Africa, with South Africa offering a more organized healthcare system including access to three free cervical cancer screenings for women, may account for this discrepancy [25]. 

We also discovered a positive association between service availability and cervical cancer screening rate. This association was also revealed by a study conducted in Western Kenya in 2018 [26]. By providing screening services directly to communities in resource-limited areas, mobile campaigns can bypass the problem of uneven distribution of resources and improve accessibility to screening. (26–27)

Regarding geographic access, based on the finding, residence, and region were associated with cervical cancer screening. What is surprising is that there was no significant difference between the distance to a health facility, healthcare facility visits, and cervical cancer screening. Another study conducted in Southwest Ethiopia had the opposite results to this study [28]. The findings indicated that women who did not have a problem with the distance from the screening center to the medical facility were 4.4 times more likely to receive cervical cancer screening services [28]. On the other hand, the results of a study conducted in Zambia in 2019 reported that cervical cancer screening promotion during women’s clinic visits helped to improve the geographic access of the visits [29]. Although we observed no significant difference in distance and short-term visits to healthcare facilities, we cannot exclude the possibility of the potential impact of geographical distance on women’s access to cervical cancer screening services.

We found a significant association between women who made their own medical decisions and increased cervical cancer screening but not with the need for permission to seek healthcare or reluctance to visit a health facility alone. Additionally, a qualitative study in Singapore indicated that empowering women to have control over their bodies could increase screening rates [30]. Taken together, it could be crucial to encourage women to make independent decisions on their own health.

The methodological strength of this study lies in how the availability of variables was determined by linking provider data with individual-level data using GPS coordinates. This innovative approach connects health facility data with demographic information to clarify the association between availability and the cervical cancer screening rate. However, because of the geographical displacement of DHS clusters, this method of assessing access to care based on distance is susceptible to bias. The study acknowledged that women might not opt for the nearest health facility for various reasons, such as service quality, provider attitudes, or challenging terrain, and accounted for errors stemming from the displacement of population clusters. This study employed a buffer zone strategy, as recommended by the DHS guidelines for GPS data, which helps reduce misclassifications owing to geographical coordinate displacement. The large and nationally representative sample size of the present study also bolstered the generalizability of the findings to the national population. This study utilized the “5 As of access” model, which permitted this study to investigate several different dimensions of access to health care to clarify the association between access to health care and cervical cancer screening.

Despite these strengths, this study has several limitations. The cross-sectional nature of this study precluded conclusions regarding causality. Additionally, the study combined KDHS data from 2014 with KSPA data from 2010, introducing potential errors owing to the temporal gap in the data collection. Although buffer zones were used to minimize classification errors, they did not account for variations in community service quality. This study defined proximity to healthcare facilities using direct geographic distance without considering factors such as topography, elevation, actual traffic conditions, or terrain. The limited number of variables collected by the 2014 KDHS also meant that data on the accommodation and acceptability dimensions were unavailable. At the same time, there may be some confounding factors, but we have not been able to adjust for them due to the limited data available for 2014 KDHS and 2010 KSPA. Last, the reliance on self-reported data from the 2014 KDHS raises concerns about recall bias, which could lead researchers to identify associations between exposure and diseases that do not exist.

## Conclusion

In conclusion, low levels of access to care across multiple dimensions (affordability, availability, geographical access, and social influence) were associated with a lower likelihood of uptaking screening services among reproductive-age Kenyan women. To boost screening rates in Kenya, it is crucial to increase insurance coverage, expand mobile screening health facilities in rural areas, enhance the availability of screening services at health facilities, and empower women to make independent healthcare decisions.

### Electronic supplementary material

Below is the link to the electronic supplementary material.

Supplementary Material 1

Supplementary Material 2

Supplementary Material 3

Supplementary Material 4

## Data Availability

The datasets that support the conclusions of this paper are included in an additional file (Additional file 1, 2, 3, 4). Since the variables in the dataset contain a great deal of content that is not relevant to this study, the additional files are all processed datasets. Additional file 1 shows the data from the 2014 Demographic and Health Survey. Additional file 2 shows the data from the 2010 Kenya Service Delivery Assessment. Additional files 3 and 4 are the number of providers in each buffer calculated after we created buffers centered on clusters. Additional file 3 is the weighted result and file 4 is the unweighted data.

## Abbreviations

GPS: Global positioning system
HIV: Human immunodeficiency virus
KDHS: Kenya Demographic and Health Survey
KNCSG: Kenya National Cancer Screening Guideline
KSPA: Kenya Service Delivery Assessment
SSA: Sub-Saharan Africa
